# Responsive population-based cohorts as platforms for characterising pathogen- and population-level infection dynamics for epidemic prevention, preparedness and response

**DOI:** 10.2807/1560-7917.ES.2025.30.25.2400255

**Published:** 2025-06-26

**Authors:** Ivonne Morales, Van Kính Nguyen, Mirna Abd El Aziz, Ayten Sultanli, Till Bärnighausen, Heiko Becher, Sandra Ciesek, Beate Kampmann, Berit Lange, Jan Rupp, Simone Scheithauer, Helen Ward, André Karch, Claudia M Denkinger

**Affiliations:** 1 Department of Infectious Disease and Tropical Medicine, Center for Infectious Diseases, Heidelberg University Hospital, Heidelberg, Germany; 2 Heidelberg Institute for Global Health (HIGH), Heidelberg University Hospital, Heidelberg, Germany; 3 School of Public Health, Imperial College London, London, United Kingdom; 4 Institute of Tropical Medicine, Travel Medicine, and Human Parasitology, University Hospital Tübingen, Tübingen, Germany; 5 Department of Global Health and Population, Harvard TH Chan School of Public Health, Harvard University, Boston, United States; 6 Africa Health Research Institute, KwaZulu-Natal, South Africa; 7 Institute for Medical Virology, University Hospital Frankfurt, Goethe University Frankfurt, Frankfurt am Main, Frankfurt, Germany; 8 German Center for Infection Research (DZIF), Frankfurt am Main, Frankfurt, Germany; 9 Fraunhofer Institute for Translational Medicine and Pharmacology ITMP, Frankfurt am Main, Frankfurt, Germany; 10 The Vaccine Centre, Faculty of Infectious and Tropical Diseases, London School of Hygiene and Tropical Medicine, London, United Kingdom; 11 Medical Research Council Unit The Gambia, London School of Hygiene and Tropical Medicine, Fajara, The Gambia; 12 Department of Epidemiology, Helmholtz Centre for Infection Research (HZI), Braunschweig, Germany; 13 German Center for Infection Research (DZIF), Braunschweig, Germany; 14 Department of Infectious Diseases and Microbiology, University Hospital Schleswig-Holstein, Campus Lübeck, Lübeck, Germany; 15 German Center for Infection Research (DZIF), Partner Hamburg-Lübeck-Borstel-Riems, Germany; 16 Department of Infection Control and Infectious Diseases, University Medical Center Göttingen (UMG), Georg-August University Göttingen, Göttingen, Germany; 17 Imperial College Healthcare NHS Trust, London, United Kingdom; 18 National Institute for Health Research Imperial Biomedical Research Centre, London, United Kingdom; 19 MRC Centre for Global Infectious Disease Analysis and Abdul Latif Jameel Institute for Disease and Emergency Analytics, Imperial College London, London, United Kingdom; 20 Institute of Epidemiology and Social Medicine, University of Münster, Albert-Schweitzer-Campus 1, Münster, Germany; 21 German Center for Infection Research (DZIF), Partner Heidelberg, Heidelberg, Germany

**Keywords:** Population-based cohort, pandemic prevention, pandemic preparedness, population panels, Germany, epidemics, cohort

## Abstract

Establishing population-based cohorts is indispensable for effective epidemic prevention, preparedness and response. Existing passive surveillance systems face limitations in their capacity to promptly provide representative data for estimating disease burden and modelling disease transmission. This perspective paper introduces a framework for establishing a dynamic and responsive nationally representative population-based cohort, with Germany as an example country. We emphasise the need for comprehensive demographic representation, innovative strategies to address participant attrition, efficient data collection and testing using digital tools, as well as novel data integration and analysis methods. Financial considerations and cost estimates for cohort establishment are discussed, highlighting potential cost savings through integration with existing research infrastructures and digital approaches. The framework outlined for creating, operating and integrating the cohort within the broader epidemiological landscape illustrates the potential of a population-based cohort to offer timely, evidence-based insights for robust public health interventions during both epidemics and pandemics, as well as during inter-epidemic periods.

## Background

Timely detection of and response to emerging pathogens is central to epidemic preparedness. [1]. To support preparedness efforts, public health surveillance systems routinely collect and analyse clinical and epidemiological indicators to understand infectious disease patterns, deriving parameters like the incubation period and the basic reproductive number (R0), used in predicting disease spread and assessing public health countermeasures [2]. Typically, the data used for these metrics come from case-based notification systems, resulting in reporting delays and incomplete population representation [2]. In addition, digital sources like social media and alert networks can provide near real-time information and may serve as a supplementary tool for disease surveillance. However, they often lack the specificity needed for decision-making [3]. To address these gaps, prospective population-based cohorts, in which a sample of the population is selected and followed over time [1], can complement routine and digital surveillance by providing a comprehensive dataset for actively identifying symptomatic, asymptomatic and minimally symptomatic individuals and their contacts. These cohorts are invaluable for understanding the characteristics of (emerging) infections, providing more accurate estimates of disease transmission parameters, death rates and clinical epidemiologic metrics [4-6]. Population-based cohorts offer substantial added value—not only during an epidemic but also in the periods between them, when they can actively contribute to prevention. For example, cohort infrastructures can be used to monitor behaviour change and emerging risk patterns (e.g. hygiene practices or social norms related to disease transmission). This makes cohorts a strategic long-term investment—not just for preparedness, but for prevention. Beyond infectious disease surveillance, cohorts can help monitor well-being and evaluate public acceptance of health measures [7], providing early insights into their effectiveness and the need for further interventions. Indeed, the COVID-19 pandemic underscored how epidemics can disrupt people’s daily lives in multiple ways, impacting their social, mental and physical well-being [8,9].

Population-based cohorts have played a crucial role in understanding the incidence, dynamics and determinants of infectious diseases, including endemic and seasonal pathogens such as influenza virus or respiratory syncytial virus (RSV), as well as emerging threats like severe acute respiratory syndrome coronavirus 2 (SARS-CoV-2). The metrics we can obtain from population-based cohorts are vital for designing and monitoring targeted public health interventions for pandemic prevention and preparedness. Examples of population-based cohorts include the UK REal-time Assessment of Community Transmission (REACT) studies, the German ELISA cohort for SARS-CoV-2, and the Latin American cohorts for influenza [4-6,10]. The REACT studies addressed key knowledge gaps during the COVID-19 pandemic. Initiated early on, they delivered timely high-resolution data on infection prevalence, transmission dynamics, emerging variants, vaccine effectiveness and geographic spread, directly informing government decisions and supporting rapid public health action, e.g. the timing of lockdowns, school closure policies and social gatherings [4,10]. 

Creation of such a cohort, however, is time-consuming and is often complicated by multiple bottlenecks. For instance, the 2015–16 Zika virus epidemic illustrated major challenges in funding, regulatory approvals, logistics and patient recruitment [11]. Although research consortia like ZIKAlliance (https://zikalliance.tghn.org) mobilised quickly, these bottlenecks delayed coordinated research efforts considerably, taking over 8 months to establish protocols. This delay meant that the peak of the epidemic had already passed in many areas before data collection could begin, severely limiting the ability to assess the full impact of Zika virus infection, particularly its association with congenital abnormalities in infants born to exposed pregnant women. An established cohort infrastructure could have enabled researchers to capture critical early-stage data, improved the precision of congenital anomaly risk estimates, and delivered timely evidence to guide public health responses and inform care for pregnant women and their families. Similarly, the development of the German National Pandemic Cohort Network (NAPKON), created to support COVID-19 research at the national level, initiated patient enrolment in the fall of 2020, once the pandemic was already well underway in the country. A key implementation challenge involved the organisation of study sites and the timely recruitment of participants, with a median time of 54 days from ethics approval to first patient enrolment. This study highlighted the critical importance of early study site preparation to minimise delays in patient recruitment [12]. These lessons underscore the need for responsive population-based cohorts as an integral part of pandemic preparedness.

In the absence of a representative cohort, a network of smaller population panels could serve as a provisional solution, which can later be ideally integrated into a robust and responsive framework. While synthesising evidence across multiple smaller population panels is possible, such efforts are often hampered by difficulties in data harmonisation, constraints on data sharing [13], and the need for methods to reconcile different study designs [13,14], limiting the power and timeliness of analyses. 

In Germany, however, the IMMUNEBRIDGE project has shown that such panels can yield timely, harmonised and model-usable estimates of SARS-CoV-2 seroprevalence [15]. The effectiveness of such a strategy in future health crises will depend on the ability to harmonise broader clinical and epidemiological outcomes, and on the adaptability of existing panels to respond swiftly to emerging pathogens, while ensuring representativeness through the inclusion of dynamic and underserved populations, such as migrants and refugees. 

An ideal population-based cohort should be agile and adaptive, capable of monitoring various infectious diseases and selectively activating or expanding as needed by time, location and changes in population composition. Moreover, it should be representative to support robust and accurate estimation of clinical and epidemiological parameters. In this Perspective, we consider methodological and financial aspects that underpin a cohort’s functionality: sample representativeness, cohort size and sampling frequency, strategies to address participant attrition, data collection and testing procedures. We present examples and discuss the challenges of establishing a long-term nationally representative cohort and possible solutions using the German experience.

## Representativeness

For a cohort to be representative of a country’s population, cohort enrolment needs to be based on random processes. In many contexts, single-stage random selection from the full population sampling frame is typically not feasible, even though it would statistically be the most efficient sampling process. Instead, higher levels of social organisation, typically geographically contiguous communities, are sampled in a first stage, before families or individuals are sampled within the randomly selected communities. These communities often coincide with first or second administrative unit levels in a country and should be considered for sampling, as national sampling frames are usually available for these units [4]. At the first administrative level, for example, Germany has 16 federal states, each with varying degrees of heterogeneity in population density and age distribution, as well as in governance structure, sociodemographic characteristics and level of urbanisation. Because age-specific patterns of infection are important for disease transmission and adaptation of interventions, the cohort should include individuals of all ages, i.e. children, adolescents, adults and elderly people, through an appropriate sampling strategy. For instance, a household-based random sampling approach (probability-based), could effectively represent participants of all ages if the sampling frame can be readily defined through resident registries or census data. An example of a population cohort employing this approach is the longitudinal cohort for influenza in Peru, which included participants from randomly selected households, ranging in age from under 2 years to over 65 years [16]. Using this strategy, the cohort achieved representativeness at the level of each of four ecologically distinct regions in the country. Alternatively, a sampling approach based on population density could be employed when up-to-date census data are lacking, as was done in Mozambique [17]. In Germany and beyond, established panels that are representative of the general population include the German Social Science Infrastructure Services (GESIS) Panel [18], the German Socioeconomic Panels (SOEP) [19], and the LISS (Longitudinal Internet studies for the Social Sciences) panel in the Netherlands [20]. However, these panels are often limited to narrower age groups, geographic coverage, or originally designed for purposes other than epidemic monitoring.

Representation of minority and dynamic populations, such as refugees and immigrants, is also essential, as these groups may be disproportionately burdened compared with the rest of the population [21]. Similar to the German Socioeconomic Panels (SOEP), targeted sampling as well as frequent refreshment sampling of minority groups, e.g. NU(M)KRAINE in Germany [22], can be conducted to ensure cohort representativeness over time [19].

## Cohort size and sampling frequency

To estimate the cohort size of a population-representative cohort, the following key parameters must be considered: (i) the level of disease prevalence with its degree of uncertainty, (ii) the expected sensitivity and specificity of the diagnostic test for case detection, (iii) the response rate and (iv) the study’s design effect [23]. The Table shows the estimated cohort sample size for Germany when these parameters are considered across various scenarios. For a detailed description of the calculations, see Supplementary File S1. These estimates are approximate and would require further refinement during implementation. The sample size can be adjusted for questions (disease prevalence in a more localised geographical region or among elderly people, for example) that either impact specific populations or exhibit higher prevalence rates.

**Table:** Example of the estimated state-level population-representative sample size in units of households or individuals, Germany

Diagnostic test characteristics		Per German state				All 16 German states	
		Target cohort size^a^		Number of households to invite^b^		Number of households^c^	
Sensitivity	Specificity	Individuals	Households	Survey 1	Survey 1+	Target	Invite
0.7	0.7	50,802	36,577	146,310	14,631	585,239	2,340,956
0.7	0.8	25,044	18,032	72,127	7,213	288,507	1,154,028
0.7	0.9	10,113	7,281	29,125	2,913	116,502	466,007
0.7	1	545	392	1,570	157	6,278	25,114
0.8	0.7	32,573	23,453	93,810	9,381	375,241	1,500,964
0.8	0.8	17,455	12,568	50,270	5,027	201,082	804,326
0.8	0.9	7,492	5,394	21,577	2,158	86,308	345,231
0.8	1	477	343	1,374	137	5,495	21,980
0.9	0.7	22,662	16,317	65,267	6,527	261,066	1,044,265
0.9	0.8	12,870	9,266	37,066	3,707	148,262	593,050
0.9	0.9	5,783	4,164	16,655	1,666	66,620	266,481
0.9	1	424	305	1,221	122	4,884	19,538

^a^ The sample size calculation of the target cohort size of individuals is based on a hypothetical emerging disease with a low prevalence of 1%, assuming a 95% confidence interval with a half-width of 1%, considering a range of typical sensitivity and specificity values of diagnostic tests, recognising that lower sensitivity or specificity requires a larger sample size. The number of target households was obtained by multiplying the target sample size of individuals by the square of the design effect factor of 1.2 to reflect the reduced statistical efficiency of household-based sampling compared to simple random sampling and divided by the number of individuals per household (considering an average of 2.0 individuals per household (2021) [43].

^b^ Survey 1 shows the number of households to invite considering an overall response rate of 25%, i.e. on average half of the invited household responded (50%), within each household half of the members decide to join (50%). Survey 1+ denotes the number of newly recruited participants needed per survey to maintain the cohort’s representativeness due to attrition and changes in the population, considering a replacement of 10% per survey.

^c^ The total number of households to invite across all 16 German federal states is calculated by multiplying the number of households to invite per state (from the Survey 1 column) by 16. Similarly, the total target number of households is obtained by multiplying the per-state household target by 16.

Defining the sampling frequency is a challenging task that requires striking a balance between disease detection at the desired prevalence level, costs and participant burden. One possible approach is to integrate representative data from a population-representative cohort with more frequently collected surveillance data, i.e. such as the Early Warning and Response System of the Robert Koch Institute (German national public health institute), into mathematical models that allow, for example, estimating changes and trends over time [24]. In this scenario, data from a population-based cohort would provide the anchor points of the true prevalence for the model estimates, whereas routinely collected surveillance data would inform model estimates of changes and trends over time.

## Response rate, participant retention, sample refreshment and rotational design

Although a response rate of 50% at either the household or individual level is used in our example in the Table, higher response rates are essential to ensure the representativeness of estimated metrics. In practice, however, lower participation rates are common. Large-scale cohort and panel studies, such as the German National Cohort (NAKO) and SOEP in Germany or the United Kingdom’s REACT cross-sectional surveys, have reported varying response rates. For instance, NAKO reported response rates ranging from 9 to 32%, depending on the recruiting study centre [25]. The SOEP has reported rates between 31.5 and 80.8%, varying by population type and wave of recruitment or refreshment sample [26]. Similarly, the REACT-1 study reported response rates ranging from 11.7 to 30.5% depending on the round of data collection [4]. Therefore, sensitisation of the population to the utility of cohorts and participation in research, as well as their engagement throughout the life of the cohort will be crucial to maximising response rates and retention. Community outreach and participant engagement through advertisement of the study, community events, healthcare providers and the media have been shown to improve participant recruitment [27]. 

Participant retention in a cohort is essential to maintaining the cohort’s representativeness. Participant attrition is, in part, influenced by age and can be intensified by the perceived burden linked to frequent sampling, insufficient incentives for involvement, or a decline in motivation and interest. Incentives, such as providing gifts or access to study results, is a common strategy often employed in survey studies to improve participant retention [19,28]. For example, cohort studies with high retention rates have reported success by using personalised retention strategies, such as providing food, transportation support, financial compensation, and consistent communication through reminders and birthday cards. These studies also emphasise the importance of a well-trained research team that is innovative, persistent and organised [29]. Additionally, a meta-analysis evaluating retention strategies in longitudinal cohort studies found that offering multiple methods of data collection, such as a combination of face-to-face and phone-based approaches, improved participant retention by 10% [30].

Participant attrition may also lead to selection bias, especially given the migration dynamics in Europe in recent years [31]. For this reason, scheduling refreshment samples over time would be essential to maintaining the representativeness of the cohort. In line with the German SOEP’s approach [19], refreshment sampling could be undertaken to increase the entire sample size or a specific subgroup of interest according to the epidemiological context. The sample can then be weighted as appropriate to ensure the representativeness of the sample. In addition, to reduce the burden on participants and increase the representativeness of the sample, a portion of the cohort could be included in the sample for a selected round of the survey and rotated (‘rotational sampling’) [32].

## Methodology for data collection and specimen collection and testing

Digital technologies have transformed cohort study methodologies, offering modern alternatives to traditional paper-based methods for data collection, communication, and participant engagement [33]. Tools such as smartphones, web-based applications, and wearable sensors facilitate remote monitoring of physiological parameters, including heart rate, respiratory rate and body temperature, while also enabling real-time symptom reporting and improving long-term participant retention [33,34]. These platforms support direct communication between participants and research staff through SMS, instant messaging, or videoconferencing, enhancing personalised engagement [33,34]. Additionally, digitalisation allows for in-depth, voice-recorded qualitative interviews, which can reveal themes beyond the scope of structured surveys [35]. Large language models such as GPT-4 (OpenAI) can further contribute by assisting in qualitative data analysis, translating languages for non-native speakers, and serving as virtual assistants to support research staff and participant communication [35].

A notable example from the German context is the 'Prospective Monitoring and Management App (PIA)', used in the ZIPCO study (Integrated DZIF Infection Cohort within the German National Cohort), which featured desirable features in digital cohort tools, such as adaptable questionnaires for various pathogens, integration with external data systems, user-friendly design, and good participant acceptance [33].

Adopting minimally invasive, decentralised and scalable sampling methods will be critical for acceptance and maximising cost-effectiveness. To adapt to different pathogens and required sampling methods, dedicated study sites must be available in all study regions that can respond and scale quickly. This would ensure that surveys can adapt to new testing methods whenever they become available for a new pathogen. In the later phases of epidemics, self-testing, i.e. with rapid antigen tests, approaches are often possible. However, these must first be validated and are often not yet available at the beginning of an epidemic.

A complementary test to pathogen detection itself is pathogen sequencing. During the COVID-19 pandemic, genomic surveillance was critical for identifying viral variants, surveying their global spread, and redefining our knowledge of disease transmission rates, health outcomes and vaccine efficacy, which ultimately determined the public health response to the pandemic [36]. In this regard, established sequencing infrastructures could provide their expertise and resources [37]. Furthermore, antibody surveillance is key to assessing population immunity and should go hand in hand with genomic surveillance.

## Infrastructure and costs

Maintaining a large longitudinal cohort is a complex logistical undertaking. Study management, sample collection and analysis, quality assurance, sample storage and transport, and sample refreshment account for most of the costs. Based on a recent cost and cost-effectiveness analysis of active surveillance testing for SARS-CoV-2 in Germany, we estimate a cost per sample of EUR 100–150, which would be ca 5–7.6 million, 2.5–3.75 million, and EUR 1–1.5 million per survey round per German federal state, if each sample were tested under a 70/70%, 70/80% or 70/90% sensitivity/specificity scenario, respectively (Table). These estimates consider the cost per participant for study management, a hotline for study participants, development of study materials, study design, IT services and postal services for shipping RT-LAMP kits, and sample analysis by RT-LAMP and confirmation by RT-qPCR [38]. Another reference is provided by the REACT study [4], which reported the cost of recruiting participants at GBP 26 (EUR 30.6) without including the cost of PCR and genomic testing (personal communication, Aidan Irwin-Singer, 18 Oct 2022). Integrating existing genomic testing platforms into the cohort [37] and methods to reduce the number of tests through pooling could help to reduce costs [39]. 

## Building a population-representative cohort and link to existing research infrastructures

Building a new long-term cohort is costly and requires a strategic approach to appropriately manage the financial and organisational costs of establishing and maintaining it. An approach that could be deemed practical for mitigating the financial and organisational burdens associated with initiating and sustaining a novel long-term cohort involves leveraging existing cohorts and extending their reach (both in geographical coverage and participant age). This adaptation would involve tailoring the study protocol and incorporating innovative sampling techniques, such as self-testing. In Germany, the SOEP [19] and the NAKO Health Study [25] are two prominent large-scale longitudinal studies with robust research frameworks. These cohorts, however, were not designed for epidemic monitoring purposes, nor were they meant to achieve national representativeness; as such, they have limited age and geographic coverage (NAKO) or lack sufficient statistical power to detect diseases at a low prevalence (SOEP). Nevertheless, the SOEP has served as a major platform for supplementary surveys amid the COVID-19 pandemic. The NAKO cohort has also been used for SARS-CoV-2 surveys and contributed samples and data to other studies [15,28,40]. Although these studies held narrower scopes, they underscored the potential of leveraging large population panels, albeit requiring intricate methodological adjustments to the sampling scheme, sample size and target population. Consideration can also be given to using the research infrastructure of these cohorts for a novel adaptive population-representative cohort, which could offer cost savings while simultaneously facilitating the creation of a more suitable and population-representative cohort.

Furthermore, while such an approach may be resource-intensive, its multi-purpose functionality and adaptability aim to maximise utility and cost-effectiveness by using only a subset of the total cohort and part of the network for repeated surveys outside of epi- or pandemic situations, such as during and after the respiratory season.

To strengthen responses to future health threats, multi-country coordination of preparedness and response capacities under a unified framework will be vital across Europe and beyond [41]. Similarly, initiatives like the EU-funded ORCHESTRA (Connecting European Cohorts to Increase Common and Effective Response To SARS-CoV-2 Pandemic) project, which connected large-scale population-based cohorts across Europe and internationally, can foster greater collaboration, facilitate data sharing, advance knowledge transfer, and guide public health decision-making [42].

## Conclusions

Adopting a population-based longitudinal strategy for active infectious disease research, supported by novel and established methods and infrastructures for data integration, decentralised testing, and communication, holds the potential to substantially transform our capacity for swift, evidence-driven and efficient responses to epidemics, pandemics and interepidemic threats.

## Data Availability

Not applicable.

## †Author’s correction

The name of Ayten Sultanli was misspelled at publication. The spelling was corrected on 16 July 2025 at the request of the authors.

## Supplementary Data

128000 Supplement
