# Author’s correction for Euro Surveill. 2025;30(25)

**DOI:** 10.2807/1560-7917.ES.2025.30.28.250717c

**Published:** 2025-07-17

**Authors:** Eurosurveillance editorial team

**Affiliations:** 1 European Centre for Disease Prevention and Control (ECDC), Stockholm, Sweden

In the article entitled ‘Responsive population-based cohorts as platforms for characterising pathogen- and population-level infection dynamics for epidemic prevention, preparedness and response’ by Morales et al., published on 26 June 2025, the name of Ayten Sultanli was misspelled at publication. The spelling was corrected on 16 July 2025 at the request of the authors.

