# Supplementary material for: Responsive population-based cohorts as platforms for characterising pathogen- and population-level infection dynamics for epidemic prevention, preparedness and response
Source: Euro Surveill. 2025 Jun 26;30(25):2400255. doi: 10.2807/1560-7917.ES.2025.30.25.2400255 (PMC12207196; doi:10.2807/1560-7917.ES.2025.30.25.2400255)
Supplement: Supplement [file 24-00255_MORALES_Supplement.pdf]

Responsive population-based cohorts as platforms for characterising pathogen- and population-level infection dynamics for epidemic preparedness and response

Disclaimer: This supplementary material is hosted by Eurosurveillance as supporting information alongside the article, on behalf of the authors, who remain responsible for the accuracy and appropriateness of the content. The same standards for ethics, copyright, attributions and permissions as for the article apply. Supplements are not edited by Eurosurveillance and the journal is not responsible for the maintenance of any links or email addresses provided therein.

Supplementary File 1.

Sample size estimate for Germany to represent the general population at the state level (16 states) considering the performance of the diagnostic test, disease prevalence, a response rate of 50% and a design effect of 1.2.

In this context, prevalence is calculated by applying a diagnostic test to a representative sample and counting the number of individuals who test positive. However, the test may be imperfect, with sensitivity or specificity less than 1. Neglecting the test's performance can result in biased estimates of the true prevalence. Similarly, calculating the sample size without accounting for test performance may lead to an underpowered study.

When the test is applied to the sample, the number of positive test results (T) in a sample of size N follows a binomial distribution:

$$T \sim \ Binom(N, \rho)$$

where ρ is the probability of a person testing positive. Thus,

$$\rho = se \times \pi \ + \ (1 - sp) \times (1 - \pi)$$

where π is the true prevalence and *se* is the test sensitivity and *sp* the test specificity. The estimate for the true prevalence is given by (Rogan and Gladen 1978):

$$\pi^* \ = \frac{\rho \ + \ sp \ - \ 1}{se \ + \ sp \ - \ 1}$$

With the variance

$$var(\pi^*) = \frac{\rho(1 - \rho)}{N \ (se \ + \ sp \ - \ 1)^2}$$

assuming that *se* and *sp* are known. For a large sample size, the prevalence can be approximated to a normal distribution with the above mean and variance. Then we can choose a sample size *n* such that

$$var(\pi^*) \ = \ (\frac{L}{Z_{\alpha/2}})^2$$

where L is the half width of the needed confidence interval and $Z_{\alpha/2}$ is the z-score of the respective confidence level. The sample size is then estimated as

$$n = (\frac{Z_{\alpha/2}}{L})^2 \frac{[se \times \pi + (1 - sp) \times (1 - \pi)] \times [1 - se \times \pi - (1 - sp)(1 - \pi)]}{(se + sp - 1)^2}$$

References

Rogan, Walter J., and Beth Gladen. 1978. 'ESTIMATING PREVALENCE FROM THE RESULTS OF A SCREENING TEST'. *American Journal of Epidemiology* 107 (1): 71–76. https://doi.org/10.1093/oxfordjournals.aje.a112510.
